# Narrow Dietary Niche With High Overlap Between Snow Leopards and Himalayan Wolves Indicates Potential for Resource Competition in Shey Phoksundo National Park, Nepal

**DOI:** 10.1002/ece3.70873

**Published:** 2025-01-21

**Authors:** Sandesh Lamichhane, Bikram Shrestha, Bhumi Prakash Chaudhary Tharu, Raj Kumar Koirala, Bishnu Prasad Bhattarai, Pratigyan Poudel, Binaya Adhikari, Gopal Khanal

**Affiliations:** ^1^ School of Forestry and Natural Resource Management, Institute of Forestry Tribhuvan University Kathmandu Nepal; ^2^ Department of Biodiversity Research, Global Change Research Institute Czech Academy of Sciences Brno Czech Republic; ^3^ Institute of Forestry Tribhuvan University Pokhara Nepal; ^4^ Central Department of Zoology, Institute of Science and Technology Tribhuvan University Kathmandu Nepal; ^5^ Ministry of Forest and Environment Bagmati Province Nepal; ^6^ Department of Biology University of Kentucky Lexington Kentucky USA; ^7^ Department of National Parks and Wildlife Conservation, Government of Nepal Nepal; ^8^ Centre for Ecological Studies Lalitpur Nepal

**Keywords:** carnivore coexistence, dietary overlap, interspecific competition, resource partitioning, scat analysis

## Abstract

Understanding species' dietary ecology and interspecific interactions is crucial for multi‐species conservation planning. In Central Asia and the Himalayas, wolves have recolonized snow leopard habitats, raising considerable concern about resource competition between these apex predators. Using micro‐histological analysis of prey species remains (e.g., hair) in their fecal samples, we determined the prey composition, dietary niche breadth, and the extent of diet overlap between these two apex predators in Shey Phoksundo National Park, Nepal. We analyzed 152 scat samples collected along 89 survey transects from April to June 2021. Our findings reveal a significant overlap in their diets (Pianka's index = 0.93), with snow leopard and wolf scats containing the remains of 11 and 10 prey species, respectively. However, the interspecific difference in prey selection was apparent, with significant deviations between observed and expected prey use indicating non‐random prey selection relative to availability: Snow leopards exhibited a higher occurrence of wild prey items in their diet (55.28%), primarily blue sheep ( *Pseudois nayaur* ) (24.83%), whereas wolves relied predominantly on domestic livestock (67.89%), with goats ( *Capra hircus* ) accounting for over one‐fourth of their diet (29.15%). Yaks ( *Bos grunniens* ) comprised a significant portion of the biomass consumed by both predators, with higher for wolves (43.68%) than snow leopards (36.47%). Overall, the narrow dietary niche breadth with high overlap indicates potential resource competition between snow leopards and wolves. However, a comprehensive understanding of resource competition will require further study on other axes of niche partitioning, including habitat and time. Nevertheless, the region's low prey richness means that, with increasing human influence, any reduction in wild prey or increase in livestock could intensify competition between snow leopards and wolves, which could have implications for livestock depredation.

## Graphical abstract

In Central Asia and the Himalayas, wolves have recolonized snow leopard habitats, raising considerable concern about resource competition between these apex predators. Using micro‐histological analysis of prey species remains (e.g., hair) in their fecal samples, we determined the prey composition, dietary niche breadth, and the extent of their diet overlap between these two apex predators in Shey Phoksundo National Park, Nepal. Snow leopards exhibited a higher occurrence of wild prey items in their diet (55.28%), whereas wolves relied predominantly on domestic livestock (67.89%). Nevertheless, the region's low prey richness means that, with increasing human influence, any reduction in wild prey or increase in livestock could intensify competition between snow leopards and wolves, which could have implications for livestock depredation.

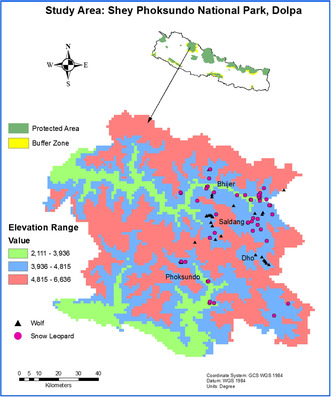

## 1 Introduction

Knowledge of what wild animal species eat, how their feeding pattern varies over space and time, and whether co‐occurring species compete for food is critical to understanding their ecology, interspecific competition and informing appropriate conservation measures (Farrell, Roman, and Sunquist 2000; Monterroso et al. 2019). While species' life history traits (Bekoff, Daniels, and Gittleman 1984) and spatiotemporal availability of food resources (Dou et al. 2019; Griffiths 1975) ultimately determine species feeding patterns, ecological theory suggests that sympatric species of similar guilds can partition the food axis, among other niches, to avoid competition for resources (Schoener 1983; Tilman 1987), thereby facilitating species coexistence and community stability (du Preez et al. 2017; Ferreiro‐Arias et al. 2021; Letten, Ke, and Fukami 2017; Müller et al. 2022; Palei, Sahu, and Nayak 2023). If food resources are limited or dominant species restrict access to specific food types, one or more sympatric species sharing the same habitat are likely to avoid consuming those resource, resulting in heterogeneity in species feeding patterns (Andheria, Karanth, and Kumar 2007; Cruz et al. 2022; Ferretti et al. 2020). As anthropogenic changes are increasingly reshaping the distribution of species and availability of food resources (Adhikari et al. 2023; Mills and Harris 2020; Wolf and Ripple 2016), understanding how such heterogeneity in species feeding patterns is produced and its implications for species interactions is theoretically motivating and practically applicable to conservation.

Large terrestrial mammalian carnivores are among the most threatened groups of species worldwide (Adhikari et al. 2022; Ripple et al. 2014). Many of these species have undergone contraction of more than 80% of their historical ranges, putting their populations at risk of local extinction (Wolf and Ripple 2017). Yet, their conservation is crucial due to their role in regulating energy flow to maintain ecosystem functioning. They also exert top‐down trophic control, preserving prey species diversity and preventing single‐species dominance while ensuring community stability (Knight et al. 2005; Sinclair et al. 2010). Information on the dietary patterns of these carnivores allows one to understand the magnitude of food competition among sympatric species and helps elucidate the food web dynamics and trophic interactions within an ecosystem (Bagchi and Mishra 2006; Palacios, Walker, and Novaro 2012). Consequently, prey selection is a widely studied aspect of carnivore ecology and biology (Hayward and Kerley 2008; Karanth and Sunquist 1995). Previous studies have investigated multiple aspects of diet, including individual species' diet composition and the impact of spatiotemporal variation in prey availability on dietary niche partitioning among sympatric carnivore species (Ramesh et al. 2012; Vissia et al. 2023). The methods for such dietary studies range from traditional micro‐histological analysis of prey remains from carnivore fecal samples to modern DNA metabarcoding and stable isotope analysis (Ferreiro‐Arias et al. 2021; Layman et al. 2007; Nielsen et al. 2018; Vissia et al. 2023).

Across the mountainous landscapes of Central Asia, the Tibetan Plateau, and the Himalayas, snow leopards ( *Panthera uncia* ) and the different sub‐species of gray wolves ( *Canis lupus* ) are the apex predators and live in sympatry. The snow leopard is categorized as vulnerable by the International Union for Conservation of Nature (IUCN) Red List and falls under Appendix 1 of the Convention on International Trade in Endangered Species (CITES), whereas the gray wolf is listed as a vulnerable species by the IUCN Red List. The global population of snow leopards is estimated to be 2700–3400 mature individuals (McCarthy et al. 2017). Wolves are believed to be more numerous than snow leopards. In particular, the species found in the mountainous regions of Nepal, India, and Bhutan is the Himalayan wolf ( *Canis lupus chanco* ), also known as the Tibetan wolf or wooly wolf. It is a subspecies of the gray wolf ( *Canis lupus* ) that has developed genetic adaptations for surviving in the cold, low‐oxygen environments of high‐altitude regions (Werhahn et al. 2018). Both species face threats from declining prey bases, increasing anthropogenic disturbance, and fragmentation in their habitat. Conflict over livestock loss is a pervasive threat to the survival of these species (Khan et al. 2018; Mishra 1997; Suryawanshi et al. 2013). While snow leopards and wolves have been believed to coexist owing to their difference in hunting behavior and habitat preferences, there has been considerable concern over the potential resource competition between them (Jumabay‐Uulu et al. 2014). In particular, wolves have recently recolonized in many areas (Subba et al. 2017), raising the possibility of competition with snow leopards and the potential impact on the behavior of snow leopard prey and blue sheep (Thapa and Rayamajhi 2023).

Multiple studies have investigated population‐level dietary patterns and prey selection for both snow leopards (Anwar et al. 2011; Aryal et al. 2014; Bagchi and Mishra 2006; Devkota, Silwal, and Kolejka 2013; Hacker et al. 2021; Lu et al. 2021; Lyngdoh et al. 2014; Oli, Taylor, and Rogers 1993; Shrestha, Aihartza, and Kindlmann 2018; Thapa et al. 2021) and Himalayan wolves (Balajeid Lyngdoh, Habib, and Shrotriya 2020; Tiralla, Holzapfel, and Ansorge 2021; Werhahn et al. 2019). More recently, many studies have also assessed diet overlap between them, highlighting the potential for resource competition (Bocci et al. 2017; Chetri, Odden, and Wegge 2017; Hacker et al. 2022; Jumabay‐Uulu et al. 2014; Kachel, Karimov, and Wirsing 2022; Pal et al. 2022a; Wang et al. 2014; Zhong et al. 2022). While these studies have broadened our understanding of carnivore diets in these regions, there is a notable research gap regarding the dietary overlap and prey selection patterns between these two species in western Nepal, where wolves have recolonized their previous habitat. Specifically, our understanding of dietary partitioning between these two apex predators in the low prey richness region like the trans‐Himalaya remains limited. Moreover, the role of livestock availability in mediating the interspecific food competition between snow leopards and wolves remains poorly studied but has implications for mitigating the consequences of human‐carnivore conflict in the region.

In this study, we assessed the dietary overlap between snow leopards and Himalayan wolves and their prey selection through microscopic analysis of prey remains in scat samples. Given the difference in their hunting ecology and behavior, the snow leopard being more specialist and wolves being more generalist, we expected that they would differ in their prey consumption and selection, with wolves having a broader dietary niche. The snow leopard, an ambush predator, favors rugged terrain and uses cliff habitats, whereas wolves, chasing predators, use rolling plains. Given these differences, we reasoned that dietary partitioning through prey species selectivity is a likely mechanism for coexistence in the study area. In particular, we hypothesized that snow leopards would consume more cliff‐dwelling species, and wolves would consume plain‐dwelling ungulates (Chetri, Odden, and Wegge 2017). By examining the diets of these two species and quantifying their overlap, this study provides insights into their ecological needs, potential for competition, and conservation requirements. Additionally, it enhances our comprehension of feeding habits and behavior in carnivore communities in high‐altitude mountain ecosystems, typically low‐productivity ecosystems with limited prey species richness, and aids authorities in developing effective conservation management strategies.

## 2 Materials and Methods

### 2.1 Study Area

**FIGURE 1:**
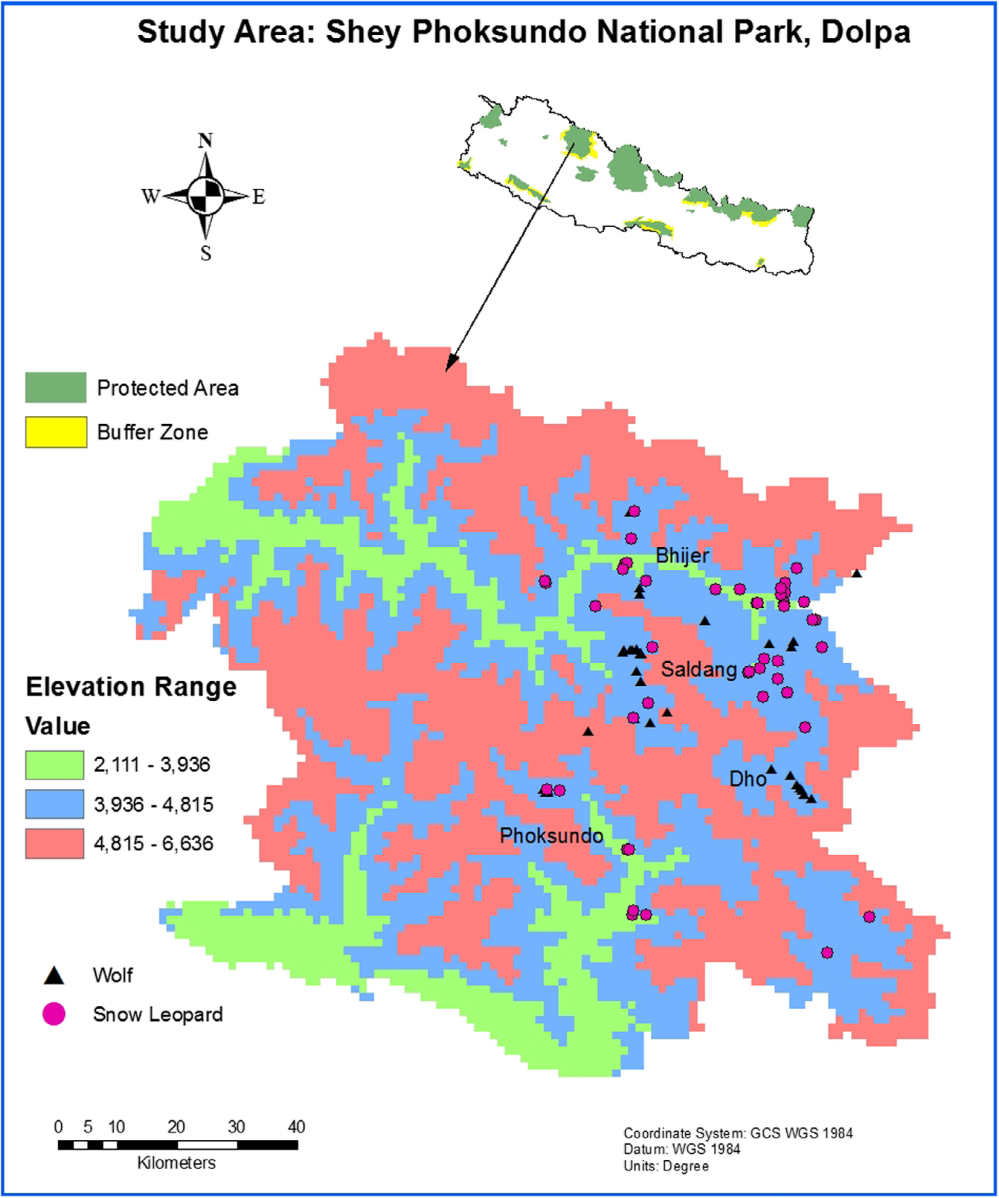
Location of wolf and snow leopard scats collected from Shey Phoksundo National Park, Nepal.

Shey Phoksundo National Park (29°21′29″ N 82°50′44″ E) lies in the Dolpa and Mugu districts, in western Nepal (Figure 1). It was established in 1984 to protect snow leopards and the trans‐Himalayan ecosystem. Its' core zone includes 3555 km^2^, and the buffer zone occupies 1349 km^2^. The park's elevation ranges from 2130 to 6883 m above the mean sea level up to the summit of Kanjiroba Mountain. This park experiences a variety of climates due to its location relative to the northern and southern aspects of the Himalayas. The Dhaulagiri and Kanjiroba Mountains act as a barrier to prevent significant rainfall from reaching the Trans‐Himalayan Region, where the main habitats for snow leopards and wolves lie. A camera trap survey conducted in Shey Phoksundo National Park and its surrounding region estimated 90 snow leopards with a population density of 2.2 individuals per 100 km^2^ (SPNP 2023). Similarly, researchers have recorded the Himalayan wolf in the central Himalayas at elevations ranging from 2937 to 5600 m (Werhahn et al. 2023).

Most precipitation occurs during the monsoon season, which lasts from mid‐June to September. The monsoon‐dominated environment in the south receives around 1500 mm of annual rainfall, whereas the dry‐dominated environment receives < 500 mm annually on the northern slopes. The winter is very harsh, with frequent snowfalls over 2500 m. The park is home to at least 36 mammal species, including snow leopard, Himalayan wolf, musk deer, Eurasian lynx, Pallas cat, leopard cat, blue sheep, jackal, Himalayan black bear, and brown bear.

### 2.2 Field Sampling

We first divided the national park into survey grids measuring 4 × 4 km square grid cells to avoid preferential sampling of some areas. We then superimposed these cells on the potential snow leopard and wolf habitat (Subba et al. 2017; WWF Nepal 2009). We individually coded each grid using alphanumeric characters to facilitate fieldwork. We selected 42 grid cells (672 km^2^) to ensure the representativeness of the snow leopard and wolves' habitat. There were four cells in the Phoksundo block, five in the Dho block, 18 in the Saldang block, and 15 in the Bhijer block. These blocks were valleys separated by high mountains, rivers, or ridgelines. The field work was conducted from spring to early summer (April to June 2021). In each grid cell, we identified potential transect locations, such as along human trails, mountain ridgelines, and valley bottoms, where the chances of detecting snow leopard and wolf signs are higher. We identified at least two transects (0.4–2.8 km long) within each grid, spaced 1.0–1.5 km apart (Jackson and Hunter 1996). The maximum number of transects per grid was three, whereas the total number of transects surveyed was 89, covering 139.2 km. We walked along these transects, looking for scats and any prey species. When encountering fresh and moderately fresh scat samples (based on odor and firmness), we collected the scats, stored them in zip‐lock bags containing silica desiccant, and labeled them properly. Fresh and moderately fresh scat samples generally have some moisture content, are dark or brown in color, and may have some odor. Scats usually < 8 days can also be categorized as ‘fresh,’ mostly used for DNA analysis (Smith et al. 2003). In contrast, old samples do not have moisture and are discolored (faded or bright white), with the outside casing often missing. Proximity of other signs, such as fresh scrapes, pugmarks, and urine, was also considered when deciding the scat samples' freshness. We left the remaining portion of scats undisturbed to avoid disrupting the communication and competition between predators (Vogt et al. 2014). We recorded the geographic coordinates, habitat type, morphological characteristics, and any indirect evidence of species presence, such as pugmarks, scrapes, etc., of the scat samples. Along the transects, we also recorded the number of prey species and livestock seen. Because we needed information on prey availability to assess the prey selection patterns of snow leopards, we recorded the abundance of all possible prey species, including blue sheep and livestock. Additionally, we also obtained data from the Shey Phoksundo National Park office regarding the number of domestic and wild prey species, which we then cross‐referenced with our field data.

### 2.3 Identification of Snow Leopards, Wolves, and Other Scat Samples

Misclassification of scat samples is one of the main problems in studies using traditional micro‐histological methods to infer the species' diet behavior, including snow leopards and wolves (Weiskopf, Kachel, and McCarthy 2016). Because we did not have the resources to genotype the scat samples, we made every effort from the start of the scat collection to avoid mislabeling one species' scat samples to another species. This happens when a researcher incorrectly labels snow leopard scat samples as wolves or other species samples or vice versa. We adopted three measures to avoid misclassification errors: First, we checked the morphological characteristics of the scat samples. Snow leopard scat samples are segmented and small in size, with fewer bones and hair, whereas wolf scats are generally unsegmented, bony, and hairy and are larger than snow leopards. We did not collect older and damaged scats, which can be properly characterized by morphological characters. The morphological characteristics of the scat samples are key to distinguishing between wolves and snow leopards. The average diameters of the scat of the snow leopards are 2.1 ± 0.5 cm (Laguardia et al. 2015), whereas the size of the wolf is 2.5–3 cm (Weaver and Fritts 1979).

Second, we scrutinized the location and habitat characteristics of the site where the scat was deposited. Snow leopards are cliff dwellers who travel along ridges with rugged terrain, whereas wolves are cursorial predators who prefer gentle slope habitats, and they defecate in areas of their higher affinity. Snow leopards' habitat includes steep terrain characterized by cliffs, ridges, gullies, and rocky outcrops, whereas wolves are found in high‐altitude scrublands on hill slopes or valley floors. After checking morphological characteristics, we assigned scats found in ridges, cliffs, steep and broken terrains, and known hills as snow leopard scats (Jackson and Hunter 1996), and those scats found in the open valleys were considered to be wolves' scats (Pal et al. 2022b).

Third, we associated the collected scat samples with nearby indirect evidence of predator presence, mainly pugmarks and scrapes. The pugmarks of snow leopards, wolves, and other sympatric species are distinct, with snow leopards having lobed oval pad shapes, whereas wolves have more elongated pugmarks with clear claw marks and symmetric digits. Wolves and other canids and predators do not leave scrapes like snow leopards. Snow leopard scrapes are clearly recognized, often with repeated marking and numerous scrapes (old and new) in relatively small areas, known as relic sites (Ahlbom and Jackson 1986). This way, only scat that could reasonably be assigned to snow leopards or wolves was collected. Scat samples likely to be of foxes, Eurasian lynx, and Pallas cats were not collected.

### 2.4 Micro‐Histological Analysis

Micro‐histological analysis is a low‐cost method used to identify species' food habits, and the process involves extracting hair remains after thoroughly washing the sample and identifying the species of origin for each hair based on its morphology (structure and characteristics) through a microscope (Putman 1984). To prepare hair slides for comparison with the reference database, we first washed all scats with cold water in a fine mesh sieve (0.5 mm). We removed non‐hair material like soil, leaves, and undigested food particles such as bones, wax, etc. The washed scats were sundried for 24 h. The dry samples were properly labeled and carefully stored in zip‐lock bags. A total of 20 hairs were extracted randomly from each sample using sieves in such a way that they could represent all kinds of hairs. Those hairs were put into a 1:1 alcohol and diethyl ether solution for 30 min. The hairs were dried at room temperature after 30 min, and five hairs were picked randomly and placed on a slide, which was then coated with transparent nail polish and let to dry. After drying up, the hair was removed from the slide, and the cast was observed. Furthermore, the hair was longitudinally sectioned to observe the medullary pattern. Those five hairs were separated for the slide and cuticle, and the medullary pattern was observed by a compound microscope under 400× magnification.

To figure out what species the hair samples come from, the cuticle and medullary patterns on the samples must be compared to known species reference collections or databases. We adopted published recommendations for the preparation of cuticle keys of prey species of snow leopards and wolves found in scats (Karanth and Sunquist 1995; Oli 1993; Shrestha, Aihartza, and Kindlmann 2018). We used a mobile phone to take photos from the compound microscope. To determine the prey species, hairs collected from the scat samples were compared with previous reference hair samples (Oli, Taylor, and Rogers 1993; Wegge, Shrestha, and Flagstad 2012). Different species of animals have distinct hair characteristics such as shape, color, diameter, scale patterns, and medullary structure (presence or absence of a central canal in the hair shaft). We also collected sample hairs from domestic goats, dogs, sheep, yaks, and horses during the field survey, from which the reference slides were prepared in the laboratory.

### 2.5 Statistical Analysis

After identifying the species for each hair sample from the scat, the statistical analysis involves counting and summarizing different metrics, such as the total number of species identified in each collected scat sample or the frequency of occurrence of any species. Because we wanted to assess the number of species represented in the scats of both snow leopards (diet breadth), we calculated the relative frequencies of occurrence of each prey species in those scats using the following formula:

Relative frequencies of occurrence of each prey species (RFO):

$$\frac{\text{Number of occurences of each food item}}{\text{Total number of occurences of}\;\mathrm{all}\;\text{food items}}\times 100$$

The occurrence of prey items in all collected scats was assessed (Mukherjee, Goyal, and Chellam 1994). Percentage frequency of occurrence and relative biomass consumed are calculated for different prey species (Ackerman, Lindzey, and Hemker 1984). Based on reference slides of hair samples, we identified the prey species identity of those five hair samples of each scat sample identified; we calculated how many times each hair sample identity occurred in total scat samples. This allowed us to compute the frequency of occurrence of each species (e.g., blue sheep), summarizing across all scat samples. Due to the abundance of hairs per unit body, the intake rate of small prey is overestimated in a carnivore's diet (Ackerman, Lindzey, and Hemker 1984; Weaver 1993). Therefore, to determine the relative proportion of biomass of various prey species consumed by snow leopards and wolves, the correction factor developed by Ackerman, Lindzey, and Hemker (1984) from feeding trials on Cougars ( *Felis concolor concolor* L.) was used. For the correction of this bias, biomass and a relative number of prey consumed were calculated using the regression equations: *Y* = 1.980 + 0.035*X* (Ackerman, Lindzey, and Hemker 1984) for snow leopards and wolves; the regression model modified by Weaver (1993) in the original version developed by (Floyd, Mech, and Jordan 1978) was used as *Y* = 0.439 + 0.008*X*. In these equations, *Y* represents the mass of mammalian prey consumed, and *X* is the mean mass of individual prey species. The mean mass of individual prey species was taken from previous studies (Lyngdoh et al. 2014; Shrestha, Aihartza, and Kindlmann 2018).

The relative biomass consumed by the sympatric carnivores was calculated using the following formula:

$$\text{Relative biomass consumed}=\frac{\text{frequencyofoccurence}\times Y}{\sum \left(\text{frequencyofoccurence}\times Y\right)}\times 100$$

Pianka's index was calculated to measure the dietary overlap between Snow Leopards and Wolves (Pianka 1974, 1973)

$$\mathrm{DO}=\frac{\sum P_{\mathrm{ij}}P_{\mathrm{ik}}}{\sqrt{\sum P_{\mathrm{ij}}^{2}P_{\mathrm{ik}}^{2}}}$$

where *P*_*ij*_ is the proportion of prey category *i* in the diet of predator *j*; *P*_*ik*_ is the proportion of prey category *i* in the diet of predator *k*. The value of dietary overlap ranges from 0 to 1, where 0 represents no overlap and 1 represents complete overlap (Pianka and Pianka 1970). We calculated the Levins index of niche breadth for both species and confidence intervals using the bootstrap method. We then compared the observed difference in the Levins index with the null distribution to test the significance of the prey selection difference between the two species. A G‐test of goodness‐of‐fit was performed to test whether observed prey usage deviated significantly from expected usage. The Bonferroni‐adjusted confidence intervals were calculated to evaluate selection ratios for each prey type. A selection ratio > 1 indicates preference, while a ratio < 1 indicates avoidance. Bonferroni‐corrected confidence intervals were used to address multiple comparisons. To account for multiple comparisons across prey species, we applied the Bonferroni correction to ensure that the overall type I error rate remains within the desired threshold (e.g., *α* = 0.05). This conservative approach helps ensure robustness in identifying significant selection or avoidance.

## 3 Results

### 3.1 Diet Niche of Snow Leopards and Wolves

Out of the total 152 scat samples collected from 89 survey transects (with an average length of 1.56 km and a total length of 139.2 km), 98 (64.47%) were identified as snow leopard scat, while the remaining 54 (35.53%) were determined to be from wolves based on morphological characteristics, surrounding habitat features, and other discernible signs (such as pugmarks and scrapes) noted in the field as outlined in the methods section. The sample size was sufficient to accurately represent the diets of both the snow leopard and the wolf (Appendices 3 and 4). We identified eleven mammalian prey species in snow leopard scats, comprising six wild prey species (blue sheep *Pseudois nayaur* , Himalayan marmot *Marmota* spp., least weasel *Mustela nivalis* , mountain weasel *Mustela altaica* , pika *Ochotona* spp., stone marten *Martes* spp.) and five domestic livestock (dog *Canis lupus familiaris* , goat *Capra hircus* , sheep *Ovis aries* , horse *Equus ferus caballus* , yak *Bos grunniens* ). In contrast, wolf scats contained the same 10 mammalian prey species, except one wild prey species, the mountain weasel. Snow leopards exhibited a slightly broader niche breadth (Levin's Index: 6.45; 95% CI = 3.34–23.42) than wolves (Levin's Index: 5.98; 95% CI = 2.91–28.50), but the difference was small and not statistically significant (*p* = 0.923).

Over half of the snow leopard (54% of 98 scats) and wolf (54% of 54 scats) samples contained only one type of prey. Among snow leopard scat samples, no more than three different prey items were found from a single sample; 42% of scats contained two prey species, and only 4% contained three prey species. In the case of wolves, a maximum of four prey species were detected in any of the 54 scat samples: 37% of the samples had two species, followed by three species in 7.41% and four species in 1.85% of scat samples. In terms of the relative frequency occurrence of different prey species in the scat samples, snow leopards consumed blue sheep the most, followed by domestic goats, while the wolf consumed domestic goats the most, followed by blue sheep (Table 1). The majority of the snow leopard's diet consisted of wild prey species (55.28%), with domestic prey species making up the remainder (44.72%). On the other hand, more than half of the wolves' diet (67.89%) was from domestic prey species, with wild prey species accounting for 32.11% (Table 1). Out of 98 snow leopard scats, 41.84% contained only wild prey species, while 30 scats (30.61%) contained only livestock species. On the other hand, nine wolves' scats (16.67%) contained exclusively wild prey species, whereas 28 wolves' scats (51.85%) contained only livestock species.

**TABLE 1:** Frequency of occurrence and relative biomass of both wild prey species and livestock species based on micro‐histological analysis of snow leopard and wolf scat samples (*n* = 152).

Species	Snow leopard (Correction factor: *Y* = 1.980 + 0.035*X*)		Wolf (Correction factor: *Y* = 0.439 + 0.008*X*)	
	Frequency of occurrence (RFO %)	Relative biomass (RB) consumed (%)	Frequency of occurrence (RFO %)	Relative biomass (RB) consumed (%)
Blue Sheep ( *Pseudois nayaur* )	24.83	20.88	9.43	6.99
Dog ( *Canis lupus familiaris* )	3.57	2.38	8.48	4.96
Domestic Goat ( *Capra hircus* )	19.9	15.87	29.15	20.48
Domestic Sheep ( *Ovis aries* )	1.53	1.15	1.85	1.23
Himalayan Marmot (*Marmota* spp.)	12.76	6.95	7.26	3.45
Horse ( *Equus ferus caballus* )	3.4	7.24	6.48	12.3
Least Weasel ( *Mustela nivalis* )	5.1	2.6	4.63	2.06
Mountain Weasel ( *Mustela altaica* )	3.57	1.88	—	—
Pika (*Ochotona* spp.)	5.61	2.78	4.33	1.87
Stone Marten (*Martes* spp.)	3.41	1.8	6.46	2.97
Yak ( *Bos grunniens* )	16.33	36.47	21.93	43.68

In terms of biomass consumed, the livestock species yak had the highest relative biomass contribution to both snow leopards' and wolves' diets, with a relatively higher contribution for wolves (43.68%) than snow leopards (36.47%). Wild prey blue sheep had the second most biomass contribution to the snow leopard diet, followed by domestic goats and other species (Table 1). However, domestic goat contribution was second highest for wolves, followed by horses and blue sheep. When comparing the relative biomass contributions of wild prey and livestock, both snow leopards and wolves relied significantly on livestock, with livestock accounting for a higher proportion of biomass consumed by wolves (83%) than by snow leopards (63%). However, wild prey contribution to their diet varied: The relative contribution of wild prey was higher for snow leopards (37%) than wolves (17%) (Table 1).

### 3.2 Prey Selection and Diet Overlap

**FIGURE 2:**
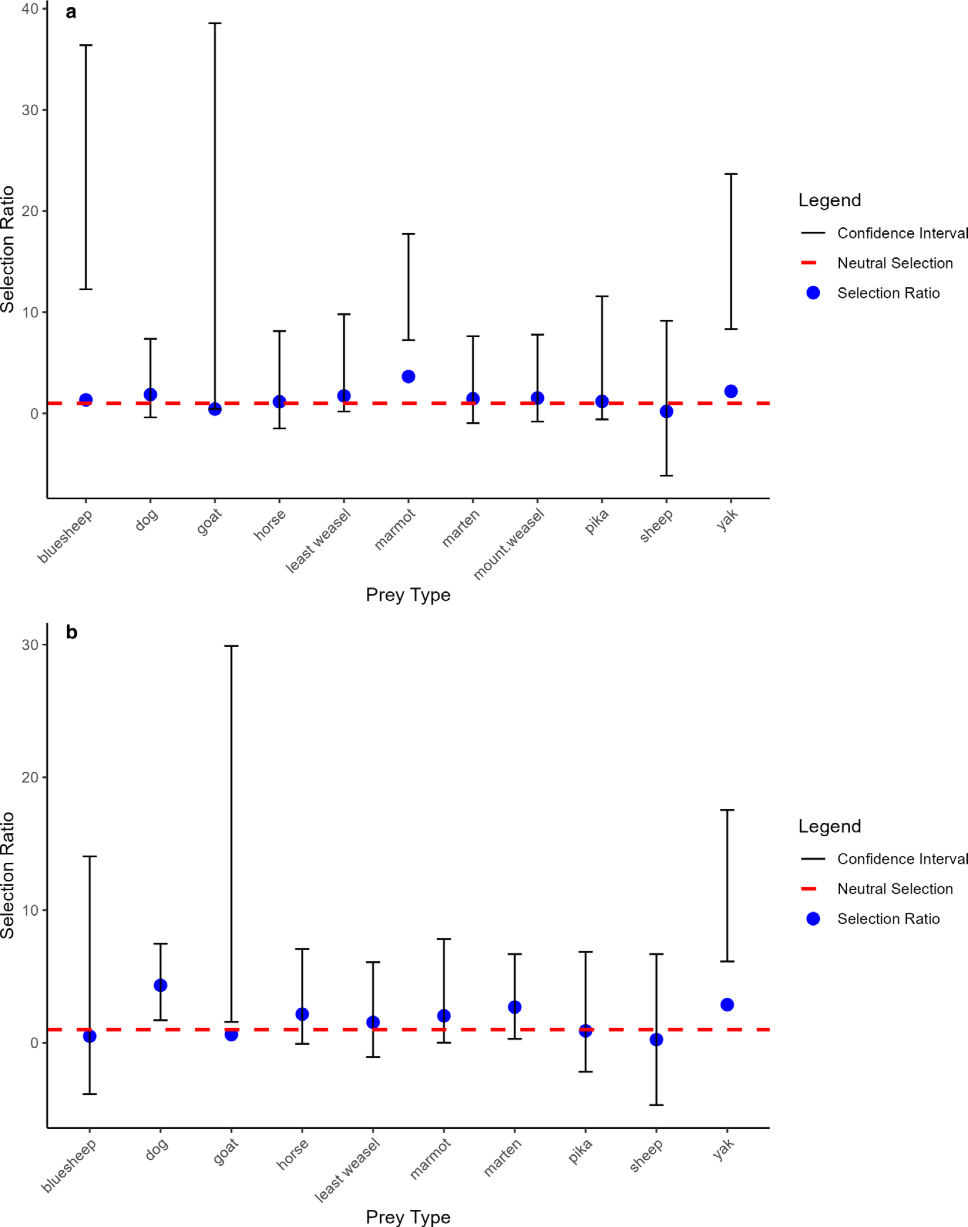
Prey Selection Ratios with Confidence Intervals. Selection ratios for (a) snow leopards and (b) wolves show prey preferences (above 1) or avoidance (below 1), with Bonferroni‐adjusted confidence intervals. The dashed line at 1 indicates a neutral selection.

Snow leopards and wolves exhibited a high diet overlap (DO) (Pianka's index=0.937). The G‐test revealed a significant deviation between observed and expected prey use by snow leopards (G = 88.62, df = 10, *p* < 0.0001) and wolves (G = 32.91, df = 9, *p* < 0.0001), indicating that prey use is not proportional to availability. Snow leopards selected blue sheep, marmots, and yaks, with no strong preference or avoidance for other prey species based on their relative availability. Wolves, on the other hand, selected yak, dog, and goat, with no strong selection for other prey species. The selection ratio for most prey species overlapped 1 with a wide confidence interval, suggesting inconclusive evidence of avoidance or selection (Figure 2, Appendix 2).

## 4 Discussion

Understanding the state of resource competition between sympatric species enables us to pinpoint limiting factors and inform conservation efforts. Dietary overlap is an important niche dimension utilized to describe the potential for resource competition and partitioning between sympatric species. Our findings indicate a significant potential for interspecific food competition between snow leopards and wolves in Shey Phoksundo National Park, Nepal. Three lines of evidence from this study suggest this is likely to be the case in our study area.

First, our analysis revealed a substantial diet overlap between these two species. Notably, the level of diet overlap we observed surpasses that reported in other studies conducted in Nepal (Chetri, Odden, and Wegge 2017; Shrestha et al. 2019), Kyrgyzstan (Jumabay‐Uulu et al. 2014), China (Hacker et al. 2022; Wang et al. 2014), Pakistan (Bocci et al. 2017), India (Justa and Lyngdoh 2023; Pal et al. 2022a), and multiple regions of Nepal and China (Werhahn et al. 2019), but is lower than reported in some parts of China (Zhong et al. 2022) and Mongolia (Kachel, Karimov, and Wirsing 2022). For instance, the recorded dietary overlaps based on Pianka's index vary from 0.44 in central Nepal (Chetri, Odden, and Wegge 2017), 0.82 in western Nepal (Shrestha et al. 2019), 0.74 in the Karakoram range in Pakistan (Bocci et al. 2017), 0.87 in the Pamirs of Northwestern China (Wang et al. 2014), to 0.91 in the Sarychat‐Ertash Reserve in Kyrgyzstan (Jumabay‐Uulu et al. 2014).

Second, we found fewer prey species in both snow leopards and wolves scats compared to other habitat, indicating potential competition for limited food resources. We identified 11 and 10 prey species from the scats of snow leopards and wolves, respectively. We note that snow leopards have a wider dietary niche than wolves, contrary to what is generally the case across their range in Asia and the Himalayas, given the generalist nature of wolves (Balajeid Lyngdoh, Habib, and Shrotriya 2020). This likely reflects the absence of plain‐dwelling ungulates like argali and Tibetan antelope, which wolves prefer in the rolling plains of Mongolia and the Tibetan plateau. Importantly, low wild prey abundance and density in the Trans‐Himalayan region indicates a constrained food supply for snow leopards and wolves (Berger, Buuveibaatar, and Mishra 2013; Singh and Milner‐Gulland 2011). In the study area, the wild prey density and richness are low (Devkota, Silwal, and Kolejka 2013). The dietary niche of snow leopards and wolves varies across different regions. In Central Nepal, snow leopards consumed 13 prey species, while wolves had 14 species in their diet (Chetri, Odden, and Wegge 2017). In India, snow leopards fed on 11 prey species, while wolves consumed 12 species (Shrotriya et al. 2022). The numbers dropped to 7 prey species for snow leopards and 5 for wolves in Pakistan (Bocci et al. 2017). Snow leopards in China have been documented consuming a wide range of 11 prey species, while wolves have been observed preying on seven different species (Wang et al. 2014).

Third, the significant reliance of wolves and snow leopards on livestock (67.89% for wolves and 44.72% for snow leopards) compared to other wild prey species suggests considerable potential for competition for food and limited wild prey availability. This level of dependence is among the highest recorded for these species in various studies conducted in different locations (Chetri, Odden, and Wegge 2017; Pal et al. 2022a; Tiralla, Holzapfel, and Ansorge 2021; Werhahn et al. 2019). While the reported relative frequency of occurrence of livestock in snow leopard scats is apparently highest in Pakistan (72%) (Anwar et al. 2011), it appears to be highest for wolves' diet (~68%) in our study area compared to other regions (Balajeid Lyngdoh, Habib, and Shrotriya 2020; Chetri, Odden, and Wegge 2017; Jumabay‐Uulu et al. 2014; Wang et al. 2014). This high dependence of both species could be due to the sheer availability of livestock whose density is two to nearly 10 times higher than wild prey (Khanal, Mishra, and Suryawanshi 2020) or could be due to lax herding practices causing greater opportunities for feeding on livestock (Jackson et al. 1996). In either case, it likely leads to greater availability of livestock as prey for snow leopards and wolves, causing them to compete over the livestock. The high reliance on livestock has implications for human‐carnivore conflict. High livestock consumption by both species can increase the risk of retaliatory killing. Reports from the study area have shown instances of retaliatory killings of snow leopards and wolves (*GK field observation*), and elsewhere as well, livestock depredation has led to retaliatory killing of both species (Nowell et al. 2016; Sonam et al. 2022).

Our results also offer insights into interspecific differences in their diet habits and preferences for prey species. Snow leopard scats primarily contained cliff‐dwelling ungulates, notably blue sheep (30.61% of snow leopard scats had blue sheep), while wolves favored plain‐dwelling ungulates, mainly livestock (46.29% and 26.62% of wolf scats had domestic goats and yaks, respectively). This prey selection reflects the species' ecology and hunting behavior, and previous studies from the study area reflect a similar pattern (Devkota, Silwal, and Kolejka 2013; Shrestha et al. 2019; Subba 2012) and also elsewhere in the region (Chetri, Odden, and Wegge 2017; Jumabay‐Uulu et al. 2014; Shrestha, Aihartza, and Kindlmann 2018; Zhong et al. 2022). However, we observed an important difference in the prey selection pattern by snow leopards and wolves compared to central Nepal (Chetri, Odden, and Wegge 2017). The relative frequency of occurence of livestock species in wolves scats was higher in our study compared to central Nepal where snow leopards' scats included a higher proportion of livestock than did wolves. Similarly, unlike the higher relative biomass contribution of marmots to wolves' diets in Central Nepal (Chetri, Odden, and Wegge 2017), we did not find evidence of wolves' substantial dependence on marmots.

Prey‐specific selection analysis revealed distinct patterns of preference and avoidance (Figure 2). The prey selection ratio and the Bonferroni confidence intervals show that snow leopards selected blue sheep, marmots, and yak and did not have significant selection for other prey species and domestic goats and sheep (Figure 2). Despite being smaller, Himalayan marmots are favored, likely due to the ease of capture and reduced energy required for hunting. Pikas had a selection ratio of 1.38, indicating occasional selection, though the overlapping confidence interval suggests a neutral or opportunistic pattern. Goats, despite their high availability, were underutilized, with a selection ratio of 0.37 and a wide confidence interval, indicating potential avoidance. Domestic sheep are not dispersed like other livestock and are guarded by their owners during grazing. Overall, this likely reflects a risk–reward trade‐off in prey acquisition by predators. The higher selection for marmots is perhaps notable; we support the previous assertion (Kachel, Karimov, and Wirsing 2022; Schaller, Junrang, and Mingjiang 1988) that the role of marmots in supporting the snow leopard population may be more important than generally believed. Instead of viewing marmots as suboptimal prey that is only available seasonally during the summer, we believe it's important to closely study the interactions between marmots, snow leopards, and wolves, especially in areas where there are no large grazing animals like Argali. This will help us better protect and monitor marmot populations, particularly the threats from disease outbreaks, eradication efforts, competition with livestock, and conflicts with pastoralists (Poudel, Spooner, and Matthews 2016). Overall, these results highlight blue sheep, yaks, and marmots as critical prey species for snow leopards. Wolves also displayed a non‐random pattern of prey selection. Yak and dog were selected more relative to availability, with no strong selection for other prey species. The high selection ratio for dogs suggests they may be an accessible or opportunistically targeted prey. This aligns with the hypothesis that human‐modified landscapes and domestic animals influence predator diets.

Although snow leopards and wolves differed in the relative contribution of prey species to their diets, with snow leopards primarily preying on wild species and wolves on livestock, the higher biomass contribution of yak in both carnivores' diets is noteworthy. This suggests that efforts by herders aimed at protecting livestock could inadvertently decrease livestock availability for both species. Consequently, this would compel them to rely more heavily on already limited wild prey abundance, thereby intensifying competition between the two predators, as has been shown elsewhere in predator–prey studies (Goodheart et al. 2021; Steinmetz et al. 2021). However, developing models to reliably predict the outcome of interactions between wolves and snow leopards and the demographic consequences for the wild prey blue sheep population will require rigorous assessments of alternative scenarios of wild prey and livestock populations.

Although we found that snow leopards and wolves are likely competing for the food resources in the study area, our results indicate some dietary partitioning between these species in terms of prey selection and relative biomass consumption. For instance, snow leopards and wolves differed in their relative consumption of wild prey vs. domestic livestock. However, our study does not establish whether the observed differences in prey selection patterns are significant enough to outweigh the dietary overlap and resulting competition over limited prey availability. While high dietary overlap may not always lead to significant exploitative competition when prey is readily available, the fact that both predators heavily rely on a small number of prey species, particularly livestock, both in terms of how frequently they appear in their diet and the amount of biomass consumed, suggests that prey availability is constrained in the area, making competition a probable outcome. Importantly, our study does not address other fundamental aspects of resource competition among sympatric species: space and time. The high dietary overlap and significant reliance of both species on livestock could mean that spatial partitioning might be facilitating co‐existence between these two species (Lovari et al. 2013). It is also likely that they are exploiting different temporal niches to avoid competition, with snow leopards exhibiting crepuscular behavior and wolves utilizing diurnal patterns as documented elsewhere (Kachel, Karimov, and Wirsing 2022; Pal et al. 2022a; Zhong et al. 2022). A full understanding of the extent of resource competition between these two species will require a concurrent assessment of their overlap over space and time because species are known to exhibit partitioning across space, time, and food.

While our study provides much‐needed information to understand the diet patterns of snow leopards and wolves and inform livestock depredation mitigation measures, we submit that our results should be considered preliminary due to the limited sample size and the short time frame (single season) of the study. Our results provide initial insights into non‐ random prey selection by both predators, but wide confidence intervals for some prey types suggest variability in predation patterns, possibly driven by environmental or behavioral factors not accounted for in this study. The snow leopard and wolves' landscapes in the Himalayas are seasonal ecosystems where wild prey and livestock availability vary across seasons. Thus, a comprehensive understanding of spatial and temporal patterns of their diet would require scat samples across seasons, which our study lacked. While a narrow dietary niche and the high overlap we documented can suggest potential competition between snow leopards and Himalayan wolves, a comprehensive understanding of prey availability, habitat dynamics, and human influences is necessary to fully grasp the complexity of food competition. Moreover, we relied on traditional visual methods to confirm snow leopard and wolf scat samples. Species confirmation through scat DNA would have improved the rigor of our study. DNA metabarcoding‐based diet analysis has emerged as powerful in studying the diet ecology of species (Ando et al. 2020); future studies would benefit from using these methods. Despite these limitations, we argue that our study provides the important information on the state of diet overlap between snow leopards and wolves in the study region, offering insights into interspecific differences in their diet useful for species conservation planning.

Our findings are particularly relevant for proactive conservation planning of these two top predators of the mountain ecosystem in Asia in the context of increasing climate change impacts on species distribution, burgeoning livestock population, and proliferating infrastructure development in their range. With the changing climate, snow leopards are likely to move to higher elevations (Aryal et al. 2016), raising concerns about their potential competition with wolf populations. Wolves are also recognizing their historic ranges, and this will likely increase their overlap with both snow leopards and livestock, increasing the potential for conflicts with local communities.

Road development continues to expand across the range of these species; habitat loss and fragmentation might push these carnivores into smaller areas, where they will compete for prey and resources. Ecological theory says that should resource competition arise between these carnivores over space and resources, the more generalist species (wolf) could outcompete the more specialized one (snow leopard). Conservation efforts thus need to consider the competitive dynamics between snow leopards and wolves amidst the changing social and ecological dynamics of the landscapes. Ensuring a sustainable prey base and mitigating competition through habitat management and prey population augmentation might be necessary.

## Author Contributions

**Sandesh Lamichhane:** conceptualization (lead), data curation (lead), formal analysis (lead), funding acquisition (lead), investigation (equal), methodology (equal), project administration (lead), resources (equal), software (equal), validation (equal), visualization (equal), writing – original draft (lead), writing – review and editing (lead). **Bikram Shrestha:** data curation (equal), formal analysis (equal), methodology (equal), software (equal), supervision (equal), validation (equal), writing – original draft (equal), writing – review and editing (equal). **Bhumi Prakash Chaudhary Tharu:** investigation (equal), project administration (equal), writing – review and editing (equal). **Raj Kumar Koirala:** data curation (equal), methodology (equal), resources (equal), supervision (lead), validation (equal), writing – original draft (equal), writing – review and editing (equal). **Bishnu Prasad Bhattarai:** methodology (equal), project administration (equal), supervision (equal), writing – original draft (equal), writing – review and editing (equal). **Pratigyan Poudel:** investigation (equal), writing – review and editing (equal). **Binaya Adhikari:** validation (equal), writing – review and editing (equal). **Gopal Khanal:** conceptualization (lead), project administration (equal), software, supervision (equal), validation (equal), visualization (lead), writing – original draft (lead), writing – review and editing (lead).

## Conflicts of Interest

The authors declare no conflicts of interest.

## Data Availability

The dataset that is associated with this study is available at Dryad (https://datadryad.org/stash/share/‐0fM0Mzp3‐PxdfHp4Jo1Nv‐kMw5ydb3EJT7QFWFcdkM).

## Appendix 1 The Abundance Estimates of Different Prey and Livestock Species Counted During the Line Transect Survey for Fecal Sample Collection and Information Available From Shey Phoksundo National Park Office, Dolpa

**Table:**

Prey type	Abundance
Dog	163
Goat	3929
Marten	200
Pika	400
Least weasel	250
Mount weasel	200
Blue sheep	1571
Yak	636
Marmot	298
Horse	250
Domestic sheep	633
Total	8530

## Appendix 2 The Selection Ratios and Bonferroni Confidence Intervals Provide Detailed Insights Into Which Prey Types Are Preferred or Avoided. A Selection Ratio > 1 Indicates Preference (Prey Is Selected More Than Expected by Availability). Less Than 1 Indicates Avoidance (Prey Is Used Less Than Expected by Availability). Confidence Intervals That Do Not Overlap 1 Confirm Significant Preference or Avoidance

**Table:**

Prey species	Selection ratio (95% CI)	
	Snow leopard	Wolf
Dog	1.87 (−0.38 to 7.38)	4.33 (1.69 to 7.47)
Goat	0.43 (0.44 to 38.56)	0.62 (1.58 to 29.91)
Marten	1.45 (−0.96 to 7.64)	2.69 (0.29 to 6.69)
Pika	1.20 (−0.58 to 11.58)	0.90 (−2.18 to 6.86)
Least weasel	1.74 (0.19 to 9.81)	1.54 (−1.07 to 6.07)
Mountain weasel	1.52 (−0.80 to 7.80)	
Blue sheep	1.35 (12.28 to 36.39)	0.50 (−3.87 to 14.05)
Yak	2.19 (8.33 to 23.67)	2.87 (6.14 to 17.54)
Marmot	3.65 (7.25 to 17.75)	2.03 (0.02 to 7.82)
Horse	1.16 (−1.48 to 8.14)	2.16 (−0.07 to 7.07)
Domestic sheep	0.21 (−6.15 to 9.15)	0.24 (−4.69 to 6.69)

## References

[ece370873-bib-0001] Ackerman, B. B. , F. G. Lindzey , and T. P. Hemker . 1984. “Cougar Food Habits in Southern Utah.” Journal of Wildlife Management 48: 147–155. 10.2307/3808462.

[ece370873-bib-0097] Adhikari, B. , K. Baral , S. Bhandari , et al. 2022. “Potential Risk Zone for Anthropogenic Mortality of Carnivores in Gandaki Province, Nepal.” Ecology and Evolution 12, no. 1: e8491. 35136552 10.1002/ece3.8491 PMC8809436

[ece370873-bib-0096] Adhikari, B. , S. C. Subedi , S. Bhandari , K. Baral , S. Lamichhane , and T. Maraseni . 2023. “Climate‐Driven Decline in the Habitat of the Endemic Spiny Babbler (*Turdoides nipalensis*).” Ecosphere 14, no. 6: e4584.

[ece370873-bib-0002] Ahlbom, G. C. , and R. M. Jackson . 1986. “Marking in Free‐Ranging Snow Leopards in West Nepal: A Preliminary Assessment.” In Proceedings of the 5th International Snow Leopard Symposium, 25–49. Srinagar, India.

[ece370873-bib-0003] Andheria, A. P. , K. U. Karanth , and N. S. Kumar . 2007. “Diet and Prey Profiles of Three Sympatric Large Carnivores in Bandipur Tiger Reserve, India.” Journal of Zoology 273: 169–175. 10.1111/j.1469-7998.2007.00310.x.

[ece370873-bib-0004] Ando, H. , H. Mukai , T. Komura , T. Dewi , M. Ando , and Y. Isagi . 2020. “Methodological Trends and Perspectives of Animal Dietary Studies by Noninvasive Fecal DNA Metabarcoding.” Environmental DNA 2: 391–406. 10.1002/EDN3.117.

[ece370873-bib-0005] Anwar, M. B. , R. Jackson , M. S. Nadeem , et al. 2011. “Food Habits of the Snow Leopard *Panthera uncia* (Schreber, 1775) in Baltistan, Northern Pakistan.” European Journal of Wildlife Research 57: 1077–1083. 10.1007/s10344-011-0521-2.

[ece370873-bib-0006] Aryal, A. , D. Brunton , W. Ji , et al. 2014. “Multipronged Strategy Including Genetic Analysis for Assessing Conservation Options for the Snow Leopard in the Central Himalaya.” Journal of Mammalogy 95: 871–881. 10.1644/13-MAMM-A-243.

[ece370873-bib-0092] Aryal, A. , U. B. Shrestha , W. Ji , et al. 2016. “Predicting the Distributions of Predator (Snow Leopard) and Prey (Blue Sheep) Under Climate Change in the Himalaya.” Ecology and Evolution 6, no. 12: 4065–4075. 10.1002/ece3.2196. 27516864 PMC4875782

[ece370873-bib-0007] Bagchi, S. , and C. Mishra . 2006. “Living With Large Carnivores: Predation on Livestock by the Snow Leopard ( *Uncia uncia* ).” Journal of Zoology 268: 217–224. 10.1111/j.1469-7998.2005.00030.x.

[ece370873-bib-0008] Balajeid Lyngdoh, S. , B. Habib , and S. Shrotriya . 2020. “Dietary Spectrum in Himalayan Wolves: Comparative Analysis of Prey Choice in Conspecifics Across High‐Elevation Rangelands of Asia.” Journal of Zoology 310: 24–33. 10.1111/jzo.12724.

[ece370873-bib-0009] Bekoff, M. , T. J. Daniels , and J. L. Gittleman . 1984. “Life History Patterns and the Comparative Social Ecology of Carnivores.” Annual Review of Ecology and Systematics 15: 191–232. 10.1146/annurev.es.15.110184.001203.

[ece370873-bib-0010] Berger, J. , B. Buuveibaatar , and C. Mishra . 2013. “Globalization of the Cashmere Market and the Decline of Large Mammals in Central Asia.” Conservation Biology 27: 679–689. 10.1111/cobi.12100. 23866036

[ece370873-bib-0011] Bocci, A. , S. Lovari , M. Z. Khan , and E. Mori . 2017. “Sympatric Snow Leopards and Tibetan Wolves: Coexistence of Large Carnivores With Human‐Driven Potential Competition.” European Journal of Wildlife Research 63: 1–9. 10.1007/S10344-017-1151-0/TABLES/2.

[ece370873-bib-0012] Chetri, M. , M. Odden , and P. Wegge . 2017. “Snow Leopard and Himalayan Wolf: Food Habits and Prey Selection in the Central Himalayas, Nepal.” PLoS One 12: e0170549. 10.1371/journal.pone.0170549. 28178279 PMC5298268

[ece370873-bib-0013] Cruz, L. R. , R. L. Muylaert , M. Galetti , and M. M. Pires . 2022. “The Geography of Diet Variation in Neotropical Carnivora.” Mammalian Genome 52: 112–128. 10.1111/MAM.12266.

[ece370873-bib-0014] Devkota, B. P. , T. Silwal , and J. Kolejka . 2013. “Prey Density and Diet of Snow Leopard (*Uncia uncia*) in Shey Phoksundo National Park, Nepal.” Applied Ecology and Environmental Sciences 1: 55–60. 10.12691/aees-1-4-4.

[ece370873-bib-0015] Dou, H. , H. Yang , J. L. D. Smith , L. Feng , T. Wang , and J. Ge . 2019. “Prey Selection of Amur Tigers in Relation to the Spatiotemporal Overlap With Prey Across the Sino–Russian Border.” Wildlife Biology 2019: 1–11. 10.2981/wlb.00508.

[ece370873-bib-0016] du Preez, B. , J. Purdon , P. Trethowan , D. W. Macdonald , and A. J. Loveridge . 2017. “Dietary Niche Differentiation Facilitates Coexistence of Two Large Carnivores.” Journal of Zoology 302: 149–156. 10.1111/JZO.12443.

[ece370873-bib-0017] Farrell, L. E. , J. Roman , and M. E. Sunquist . 2000. “Dietary Separation of Sympatric Carnivores Identified by Molecular Analysis of Scats.” Molecular Ecology 9: 1583–1590. 10.1046/j.1365-294X.2000.01037.x. 11050553

[ece370873-bib-0018] Ferreiro‐Arias, I. , J. Isla , P. Jordano , and A. Benítez‐López . 2021. “Fine‐Scale Coexistence Between Mediterranean Mesocarnivores Is Mediated by Spatial, Temporal, and Trophic Resource Partitioning.” Ecology and Evolution 11: 15520–15533. 10.1002/ECE3.8077. 34824772 PMC8601891

[ece370873-bib-0019] Ferretti, F. , S. Lovari , M. Lucherini , M. Hayward , and P. A. Stephens . 2020. “Only the Largest Terrestrial Carnivores Increase Their Dietary Breadth With Increasing Prey Richness.” Mammal Review 50: 291–303. 10.1111/mam.12197.

[ece370873-bib-0020] Floyd, T. J. , L. D. Mech , and P. A. Jordan . 1978. “Relating Wolf Scat Content to Prey Consumed.” Journal of Wildlife Management 42, no. 3: 528. 10.2307/3800814.

[ece370873-bib-0021] Goodheart, B. , S. Creel , M. S. Becker , et al. 2021. “Low Apex Carnivore Density Does Not Release a Subordinate Competitor When Driven by Prey Depletion.” Biological Conservation 261: 109273. 10.1016/j.biocon.2021.109273.

[ece370873-bib-0022] Griffiths, D. 1975. “Prey Availability and the Food of Predators.” Ecology 56: 1209–1214. 10.2307/1936161.

[ece370873-bib-0023] Hacker, C. E. , W. Cong , Y. Xue , et al. 2022. “Dietary Diversity and Niche Partitioning of Carnivores Across the Qinghai–Tibetan Plateau of China Using DNA Metabarcoding.” Journal of Mammalogy 103: 1005–1018. 10.1093/JMAMMAL/GYAC044.

[ece370873-bib-0024] Hacker, C. E. , M. Jevit , S. Hussain , et al. 2021. “Regional Comparison of Snow Leopard ( *Panthera uncia* ) Diet Using DNA Metabarcoding.” Biodiversity and Conservation 30: 797–817. 10.1007/s10531-021-02118-6.

[ece370873-bib-0025] Hayward, M. W. , and G. I. H. Kerley . 2008. “Prey Preferences and Dietary Overlap Amongst Africa's Large Predators.” African Journal of Wildlife Research 38: 93–108. 10.3957/0379-4369-38.2.93.

[ece370873-bib-0026] Jackson, R. M. , G. Ahlborn , M. Gurung , and S. B. Ale . 1996. “Reducing Livestock Depredation Losses in the Nepalese Himalaya.” In Proceedings of the Seventeenth Vertebrate Pest Conference, 241–247. Rohnert Park, California: University of California, Davis. 10.1017/CBO9781107415324.004.

[ece370873-bib-0027] Jackson, R. M. , and D. O. Hunter . 1996. Snow Leopard Survey and Conservation Handbook. Seattle, Washington and Colorado: International Snow Leopard Trust and U.S. Geological Survey, Fort Collins Science Center.

[ece370873-bib-0029] Jumabay‐Uulu, K. , P. Wegge , C. Mishra , and K. Sharma . 2014. “Large Carnivores and Low Diversity of Optimal Prey: A Comparison of the Diets of Snow Leopards Panthera Uncia and Wolves Canis Lupus in Sarychat‐Ertash Reserve in Kyrgyzstan.” Oryx 48, no. 4: 529–535. 10.1017/S0030605313000306.

[ece370873-bib-0030] Justa, P. , and S. Lyngdoh . 2023. “Understanding Carnivore Interactions in a Cold Arid Trans‐Himalayan Landscape: What Drives Co‐Existence Patterns Within Predator Guild Along Varying Resource Gradients?” Ecology and Evolution 13: e10040. 10.1002/ECE3.10040. 37181213 PMC10173057

[ece370873-bib-0031] Kachel, S. M. , K. Karimov , and A. J. Wirsing . 2022. “Predator Niche Overlap and Partitioning and Potential Interactions in the Mountains of Central Asia.” Journal of Mammalogy 103: 1019–1029. 10.1093/jmammal/gyac026.

[ece370873-bib-0032] Karanth, K. , and M. Sunquist . 1995. “Prey Selection by Tiger, Leopard and Dhole in Tropical Forests.” Journal of Animal Ecology 64, no. 4: 439. 10.2307/5647.

[ece370873-bib-0033] Khan, M. Z. , B. Khan , M. S. Awan , and F. Begum . 2018. “Livestock Depredation by Large Predators and Its Implications for Conservation and Livelihoods in the Karakoram Mountains of Pakistan.” Oryx 52: 519–525. 10.1017/S0030605316001095.

[ece370873-bib-0034] Khanal, G. , C. Mishra , and K. Ramesh Suryawanshi . 2020. “Relative Influence of Wild Prey and Livestock Abundance on Carnivore‐Caused Livestock Predation.” Ecology and Evolution 10: 11787–11797. 10.1002/ece3.6815. 33145001 PMC7593152

[ece370873-bib-0035] Knight, T. M. , M. W. McCoy , J. M. Chase , K. A. McCoy , and R. D. Holt . 2005. “Trophic Cascades Across Ecosystems.” Nature 437: 880–883. 10.1038/nature03962. 16208370

[ece370873-bib-0036] Laguardia, A. , J. Wang , F. L. Shi , K. Shi , and P. Riordan . 2015. “Species Identification Refined by Molecular Scatology in a Community of Sympatric Carnivores in Xinjiang, China.” Zoological Research 36: 72–78. 25855225 10.13918/j.issn.2095-8137.2015.2.72 PMC4790252

[ece370873-bib-0037] Layman, C. A. , D. A. Arrington , C. G. Montaña , and D. M. Post . 2007. “Can Stable Isotope Ratios Provide for Community‐Wide Measures of Trophic Structure?” Ecology 88: 42–48. 10.1890/0012-9658(2007)88[42:CSIRPF]2.0.CO;2. 17489452

[ece370873-bib-0038] Letten, A. D. , P. J. Ke , and T. Fukami . 2017. “Linking Modern Coexistence Theory and Contemporary Niche Theory.” Ecological Monographs 87: 161–177. 10.1002/ecm.1242.

[ece370873-bib-0039] Lovari, S. , I. Minder , F. Ferretti , N. Mucci , E. Randi , and B. Pellizzi . 2013. “Common and Snow Leopards Share Prey, but Not Habitats: Competition Avoidance by Large Predators?” Journal of Zoology 291: 127–135. 10.1111/jzo.12053.

[ece370873-bib-0040] Lu, Q. , L. Xiao , C. Cheng , Z. Lu , J. Zhao , and M. Yao . 2021. “Snow Leopard Dietary Preferences and Livestock Predation Revealed by Fecal DNA Metabarcoding: No Evidence for Apparent Competition Between Wild and Domestic Prey.” Frontiers in Ecology and Evolution 9: 783546. 10.3389/fevo.2021.783546.

[ece370873-bib-0041] Lyngdoh, S. , S. Shrotriya , S. P. Goyal , H. Clements , M. W. Hayward , and B. Habib . 2014. “Prey Preferences of the Snow Leopard ( *Panthera uncia* ): Regional Diet Specificity Holds Global Significance for Conservation.” PLoS One 9: e88349. 10.1371/journal.pone.0088349. 24533080 PMC3922817

[ece370873-bib-0043] McCarthy, M. D. , R. Jackson , P. Zahler , and K. McCarthy . 2017. *Panthera uncia*, Snow Leopard. The IUCN Red List of Threatened Species 8235, 27.

[ece370873-bib-0044] Mills, K. L. , and N. C. Harris . 2020. “Humans Disrupt Access to Prey for Large African Carnivores.” eLife 9: e60690. 10.7554/eLife.60690. 33206047 PMC7673783

[ece370873-bib-0045] Mishra, C. 1997. “Livestock Depredation by Large Carnivores in the Indian Trans‐Himalaya: Conflict Perceptions and Conservation Prospects.” Environmental Conservation 24: 338–343. 10.1017/S0376892997000441.

[ece370873-bib-0046] Monterroso, P. , R. Godinho , T. Oliveira , et al. 2019. “Feeding Ecological Knowledge: The Underutilised Power of Faecal DNA Approaches for Carnivore Diet Analysis.” Mammal Review 49: 97–112. 10.1111/mam.12144.

[ece370873-bib-0047] Mukherjee, S. , S. P. Goyal , and R. Chellam . 1994. “Standardisation of Scat Analysis Techniques for Leopard (*Panthera pardus*) in Gir National Park, Western India.” Mammalia 58: 139–144. 10.1515/mamm.1994.58.1.139.

[ece370873-bib-0048] Müller, L. , W. D. Briers‐Louw , R. Amin , C. S. Lochner , and A. J. Leslie . 2022. “Carnivore Coexistence Facilitated by Spatial and Dietary Partitioning and Fine‐Scale Behavioural Avoidance in a Semi‐Arid Ecosystem.” Journal of Zoology 317: 114–128. 10.1111/JZO.12964.

[ece370873-bib-0049] Nielsen, J. M. , E. L. Clare , B. Hayden , M. T. Brett , and P. Kratina . 2018. “Diet Tracing in Ecology: Method Comparison and Selection.” Methods in Ecology and Evolution 9: 278–291. 10.1111/2041-210X.12869.

[ece370873-bib-0050] Nowell, K. , J. Li , M. Paltsyn , and R. K. Sharma . 2016. An Ounce of Prevention: Snow Leopard Crime Revisited.

[ece370873-bib-0051] Oli, M. , I. Taylor , and D. Rogers . 1993. “Diet of the Snow Leopard (*Panthera uncia*) in the Annapurna Conservation Area, Nepal.” Journal of Zoology 231: 365–370.

[ece370873-bib-0052] Oli, M. K. 1993. “A Key for the Identification of the Hair of Mammals of a Snow Leopard ( *Panthera uncia* ) Habitat in Nepal.” Journal of Zoology 231: 71–93. 10.1111/j.1469-7998.1993.tb05354.x.

[ece370873-bib-0053] Pal, R. , A. Panwar , S. P. Goyal , and S. Sathyakumar . 2022a. “Changes in Ecological Conditions May Influence Intraguild Competition: Inferring Interaction Patterns of Snow Leopard With Co‐Predators.” PeerJ 10: e14277. 10.7717/PEERJ.14277/SUPP-6. 36312761 PMC9615993

[ece370873-bib-0054] Pal, R. , A. Panwar , S. P. Goyal , and S. Sathyakumar . 2022b. “Space Use by Woolly Wolf Canis Lupus Chanco in Gangotri National Park, Western Himalaya, India.” Frontiers in Ecology and Evolution 9: 782339. 10.3389/FEVO.2021.782339/BIBTEX.

[ece370873-bib-0055] Palacios, R. , R. S. Walker , and A. J. Novaro . 2012. “Differences in Diet and Trophic Interactions of Patagonian Carnivores Between Areas With Mostly Native or Exotic Prey.” Mammalian Biology 77: 183–189. 10.1016/j.mambio.2012.01.001.

[ece370873-bib-0056] Palei, H. S. , H. K. Sahu , and A. K. Nayak . 2023. “Competition Versus Opportunism: Diet and Trophic Niche Relationship of Two Sympatric Apex Carnivores in a Tropical Forest.” Acta Ecologica Sinica 43: 99–105. 10.1016/j.chnaes.2021.10.004.

[ece370873-bib-0098] Pianka, E. R. , and H. D. Pianka . 1970. “The Ecology of *Moloch horridus* (Lacertilia: Agamidae) in Western Australia.” Copeia: 90–103.

[ece370873-bib-0057] Pianka, E. R. 1973. “The Structure of Lizard Communities.” Annual Review of Ecology and Systematics 4: 53–74. 10.1146/annurev.es.04.110173.000413.

[ece370873-bib-0058] Pianka, E. R. 1974. “Niche Overlap and Diffuse Competition.” Proceedings of the National Academy of Sciences of the United States of America 71: 2141–2145. 10.1073/pnas.71.5.2141. 4525324 PMC388403

[ece370873-bib-0059] Poudel, B. S. , P. G. Spooner , and A. Matthews . 2016. “Pastoralist Disturbance Effects on Himalayan Marmot Foraging and Vigilance Activity.” Ecological Research 31: 93–104. 10.1007/S11284-015-1315-X/TABLES/4.

[ece370873-bib-0060] Putman, R. J. 1984. “Facts From Faeces.” Mammal Review 14: 79–97. 10.1111/j.1365-2907.1984.tb00341.x.

[ece370873-bib-0061] Ramesh, T. , R. Kalle , K. Sankar , and Q. Qureshi . 2012. “Spatio‐Temporal Partitioning Among Large Carnivores in Relation to Major Prey Species in Western Ghats.” Journal of Zoology 287: 269–275. 10.1111/J.1469-7998.2012.00908.X.

[ece370873-bib-0062] Ripple, W. J. , J. A. Estes , R. L. Beschta , et al. 2014. “Status and Ecological Effects of the World's Largest Carnivores.” Science 343: 1241484. 10.1126/science.1241484. 24408439

[ece370873-bib-0063] Schaller, G. B. , R. Junrang , and Q. Mingjiang . 1988. “Status of the Snow Leopard *Panthera uncia* in Qinghai and Gansu Provinces, China.” Biological Conservation 45: 179–194. 10.1016/0006-3207(88)90138-3.

[ece370873-bib-0064] Schoener, T. W. 1983. “Field Experiments on Interspecific Competition.” American Naturalist 122: 240–285. 10.1086/284133.

[ece370873-bib-0065] Shrestha, A. , K. Thapa , S. A. Subba , et al. 2019. “Cats, Canines, and Coexistence: Dietary Differentiation Between the Sympatric Snow Leopard and Grey Wolf in the Western Landscape of Nepal Himalaya.” Journal of Threatened Taxa 11: 13815–13821. 10.11609/jott.4217.11.7.13815-13821.

[ece370873-bib-0066] Shrestha, B. , J. Aihartza , and P. Kindlmann . 2018. “Diet and Prey Selection by Snow Leopards in the Nepalese Himalayas.” PLoS One 13: e0206310. 10.1371/JOURNAL.PONE.0206310. 30517109 PMC6281286

[ece370873-bib-0067] Shrotriya, S. , H. S. Reshamwala , S. Lyngdoh , Y. V. Jhala , and B. Habib . 2022. “Feeding Patterns of Three Widespread Carnivores—The Wolf, Snow Leopard, and Red Fox—In the Trans‐Himalayan Landscape of India.” Frontiers in Ecology and Evolution 10: 815996. 10.3389/FEVO.2022.815996/BIBTEX.

[ece370873-bib-0068] Sinclair, A. R. E. , K. Metzger , J. S. Brashares , et al. 2010. “Trophic Cascades in African Savanna: Serengeti as a Case Study.” In Trophic Cascades: Predators, Prey, and the Changing Dynamics of Nature, edited by J. Terborgh and J. Estes , 255–274. Washington, DC: Island Press.

[ece370873-bib-0069] Singh, N. J. , and E. J. Milner‐Gulland . 2011. “Monitoring Ungulates in Central Asia: Current Constraints and Future Potential.” Oryx 45: 38–49. 10.1017/S0030605310000839.

[ece370873-bib-0070] Smith, D. A. , K. Ralls , A. Hurt , et al. 2003. “Detection and Accuracy Rates of Dogs Trained to Find Scats of San Joaquin Kit Foxes ( *Vulpes macrotis mutica* ).” Animal Conservation 6, no. 4: 339–346. 10.1017/S136794300300341X.

[ece370873-bib-0071] Sonam, K. , R. Dorjay , M. Khanyari , et al. 2022. “A Community‐Based Conservation Initiative for Wolves in the Ladakh Trans‐Himalaya, India.” Frontiers in Ecology and Evolution 10: 809817. 10.3389/FEVO.2022.809817/BIBTEX.

[ece370873-bib-0094] SPNP . 2023. Population Assessment of Shey ‐ Phoksundo National Park's Snow Leopard and Prey, pp. 1–64, Dolpa, Nepal.

[ece370873-bib-0072] Steinmetz, R. , N. Seuaturien , P. Intanajitjuy , P. Inrueang , and K. Prempree . 2021. “The Effects of Prey Depletion on Dietary Niches of Sympatric Apex Predators in Southeast Asia.” Integrative Zoology 16: 19–32. 10.1111/1749-4877.12461. 32627329

[ece370873-bib-0073] Subba, S. A. 2012. Assessing the Genetic Status, Distribution, Prey Selection and Conservation Issues of Himalayan Wolf (Canis himalayensis) in Trans‐Himalayan Dolpa, Nepal. Lund, Sweden: Lund University.

[ece370873-bib-0074] Subba, S. A. , A. K. Shrestha , K. Thapa , et al. 2017. “Distribution of Grey Wolves Canis Lupus Lupus in the Nepalese Himalaya: Implications for Conservation Management.” Oryx 51: 403–406. 10.1017/S0030605316000296.

[ece370873-bib-0075] Suryawanshi, K. R. , Y. V. Bhatnagar , S. Redpath , and C. Mishra . 2013. “People, Predators and Perceptions: Patterns of Livestock Depredation by Snow Leopards and Wolves.” Journal of Applied Ecology 50: 550–560. 10.1111/1365-2664.12061.

[ece370873-bib-0076] Thapa, K. , and S. Rayamajhi . 2023. “Anti‐Predator Strategies of Blue Sheep (Naur) Under Varied Predator Compositions: A Comparison of Snow Leopard‐Inhabited Valleys With and Without Wolves in Nepal.” Wildlife Research 51: WR23012. 10.1071/WR23012.

[ece370873-bib-0077] Thapa, K. , N. Schmitt , N. M. B. Pradhan , H. R. Acharya , and S. Rayamajhi . 2021. “No Silver Bullet? Snow Leopard Prey Selection in Mt. Kangchenjunga, Nepal.” Ecology and Evolution 11: 16413–16425. 10.1002/ECE3.8279. 34938445 PMC8668728

[ece370873-bib-0078] Tilman, D. 1987. “The Importance of the Mechanisms of Interspecific Competition.” American Naturalist 129: 769–774. 10.1086/284672.

[ece370873-bib-0079] Tiralla, N. , M. Holzapfel , and H. Ansorge . 2021. “Feeding Ecology of the Wolf (*Canis lupus*) in a Near‐Natural Ecosystem in Mongolia.” Mammalian Biology 101: 83–89. 10.1007/S42991-020-00093-Z/TABLES/1.

[ece370873-bib-0080] Vissia, S. , F. A. S. Virtuoso , A. Bouman , and F. van Langevelde . 2023. “Seasonal Variation in Prey Preference, Diet Partitioning and Niche Breadth in a Rich Large Carnivore Guild.” African Journal of Ecology 61: 141–152. 10.1111/AJE.13098.

[ece370873-bib-0095] Vogt, K. , F. Zimmermann , M. Kölliker , and U. Breitenmoser . 2014. “Scent‐Marking Behaviour and Social Dynamics in a Wild Population of Eurasian *Lynx lynx* lynx.” Behavioural Processes 106: 98–106. 24814909 10.1016/j.beproc.2014.04.017

[ece370873-bib-0081] Wang, J. , A. Laguardia , P. J. Damerell , P. Riordan , and K. Shi . 2014. “Dietary Overlap of Snow Leopard and Other Carnivores in the Pamirs of Northwestern China.” Chinese Science Bulletin 59: 3162–3168. 10.1007/s11434-014-0370-y.

[ece370873-bib-0082] Weaver, J. L. 1993. “Refining the Equation for Interpreting Prey Occurrence in Gray Wolf Scats.” Journal of Wildlife Management 57, no. 3: 534. 10.2307/3809278.

[ece370873-bib-0083] Weaver, J. L. , and S. H. Fritts . 1979. “Comparison of Coyote and Wolf Scat Diameters.” Journal of Wildlife Management 43: 786–788. 10.2307/3808765.

[ece370873-bib-0084] Wegge, P. , R. Shrestha , and Ø. Flagstad . 2012. “Snow Leopard Panthera Uncia Predation on Livestock and Wild Prey in a Mountain Valley in Northern Nepal: Implications for Conservation Management.” Wildlife Biology 18: 131–141. 10.2981/11-049.

[ece370873-bib-0085] Weiskopf, S. R. , S. M. Kachel , and K. P. McCarthy . 2016. “What Are Snow Leopards Really Eating? Identifying Bias in Food‐Habit Studies.” Wildlife Society Bulletin 40: 233–240. 10.1002/WSB.640.

[ece370873-bib-0086] Werhahn, G. , N. Kusi , X. Li , et al. 2019. “Himalayan Wolf Foraging Ecology and the Importance of Wild Prey.” Global Ecology and Conservation 20: e00780. 10.1016/J.GECCO.2019.E00780.

[ece370873-bib-0087] Werhahn, G. , H. Senn , M. Ghazali , et al. 2018. “The Unique Genetic Adaptation of the Himalayan Wolf to High‐Altitudes and Consequences for Conservation.” Global Ecology and Conservation 16: e00455. 10.1016/J.GECCO.2018.E00455.

[ece370873-bib-0093] Werhahn, G. , L. Hennelly , S. Lyngdoh , B. Habib , S. K. Viranta , and S. Shrotriya . 2023. Himalayan Wolf: Canis lupus ssp. chanco. IUCN ‐ The World Conservation Union International Union for Conservation of Nature. https://www.iucnredlist.org/fr/species/223987824/223987841#taxonomy.

[ece370873-bib-0088] Wolf, C. , and W. J. Ripple . 2016. “Prey Depletion as a Threat to the World's Large Carnivores.” Royal Society Open Science 3: 160252. 10.1098/rsos.160252. 27853599 PMC5108949

[ece370873-bib-0089] Wolf, C. , and W. J. Ripple . 2017. “Range Contractions of the World's Large Carnivores.” Royal Society Open Science 4: 170052. 10.1098/rsos.170052. 28791136 PMC5541531

[ece370873-bib-0090] WWF Nepal . 2009. Estimating Snow Leopard Populations in the Nepal Himalaya. Kathmandu, Nepal: WWF Nepal.

[ece370873-bib-0091] Zhong, H. , F. Li , J. J. Díaz‐Sacco , and K. Shi . 2022. “Dietary and Temporal Partitioning Facilitates Coexistence of Sympatric Carnivores in the Everest Region.” Ecology and Evolution 12: e9531. 10.1002/ECE3.9531. 36440311 PMC9682211

